# Effectiveness of two antifolate prophylactic strategies against malaria in HIV-positive pregnant women in Bangui, Central African Republic: study protocol for a randomized controlled trial (MACOMBA)

**DOI:** 10.1186/1745-6215-14-255

**Published:** 2013-08-14

**Authors:** Alexandre Manirakiza, Abdoulaye Sepou, Eugène Serdouma, Samuel Gondje, Ghislain Géraud Banthas Bata, Sandrine Moussa, Aude Boulay, Jean Methode Moyen, Olga Sakanga, Lenaig Le-Fouler, Mirdad Kazanji, Muriel Vray

**Affiliations:** 1 Institut Pasteur of Bangui, International Network of Instituts Pasteur, PO Box 923, Pasteur Avenue, Bangui, Central African Republic; 2 Institut Pasteur of Paris, Unité d'Epidémiologie des Maladies Emergentes, 25 Rue du Dr Roux, 75724 Paris CEDEX 15, France; 3 Hôpital Communautaire of Bangui, Ministry of Public Health, Population and AIDS Control, PO Box 1383, Bangui, Central African Republic; 4 Hôpital de l’Amitié, Ministry of Public Health, Population and AIDS Control, PO Box 1383, Bangui, Central African Republic; 5 Maternité de la Gendarmerie, Ministry of Public Health, Population and AIDS Control, PO Box 1383, Bangui, Central African Republic; 6 Maternité du Centre de Santé des Castors, Ministry of Public Health, Population and AIDS Control, PO Box 1383, Bangui, Central African Republic; 7 Malaria Programme Division, Ministry of Public Health, Population and AIDS Control, PO Box 883, Bangui, Central African Republic

**Keywords:** Malaria prevention, HIV, Pregnancy, Antifolate

## Abstract

**Background:**

Co-infection with malaria parasite and HIV is an emerging public health problem in tropical areas, particularly in pregnant women, and management of the concurrent effects of these two infections is challenging. Co-trimoxazole is a sulfamide preparation used to prevent opportunistic infections in HIV-infected patients, and many studies have reported that it has significant activity against malaria. As the efficacy of intermittent preventive treatment (IPT) with sulfadoxine-pyrimethamine (SP) against malaria is decreasing, co-trimoxazole might be an alternative for preventing malaria among HIV-infected populations. The aim of this study is to compare the effectiveness of SP-IPT, which is recommended for the prevention of malaria during pregnancy in the Central African Republic, with that of a daily dose of co-trimoxazole against *P. falciparum* infections among HIV-infected pregnant women in Bangui, the capital of the Central African Republic.

**Methods/Design:**

The MACOMBA study (MAternity and COntrol of Malaria-HIV co-infection in BAngui) is a multicentre open-label randomized clinical trial conducted at four maternity hospitals in Bangui. All HIV-infected pregnant women presenting for an antenatal clinic visit between the weeks 16 and 28 of amenorrhoea, with a CD4 count of more than 350 cells/mm^3^, will be eligible. All the women will provide written consent before being enrolled in the study and will then be randomly allocated to either SP-IPT (25 mg of sulfadoxine and 1.25 mg of pyrimethamine) or daily co-trimoxazole doses (960 mg per dose). The primary end-point is the placental malaria parasitaemia rate at delivery. Other main outcome measures include the number of malaria episodes during pregnancy, safety, and treatment compliance. Furthermore, the frequency of molecular resistance markers *dhfr* and *dhps* will be measured.

**Discussion:**

In this trial, we seek to confirm whether co-trimoxazole is operationally suitable to replace SP-IPT in order to prevent malaria among pregnant women infected with HIV in the Central African Republic.

**Trial registration:**

ClinicalTrials.gov Identifier: NCT01746199.

## Background

Malaria and HIV infection are important global public health problems. In 2008, malaria affected more than 2 billion people, representing 40% of the world’s population. That same year, some 247 million episodes of malaria were recorded, in addition to almost 1 million deaths, of which nearly 20% were children. Each year, more than 50 million women living in malaria-endemic regions become pregnant, 20% for the first time, and more than half develop complications. In 2008, an estimated 33.4 million people were living with HIV infection, and 2 million people, including 280,000 children, died from AIDS-related diseases. Sub-Saharan Africa is the most severely affected region: in 2007, it was estimated to contain 67% of all people living with HIV in the world, 75% of all women infected with HIV, and 72% of all deaths from AIDS [1]. These two endemic diseases are thus a double public health problem on the African continent [2]. The interaction between HIV infection and malaria was first described in 1992 [3], and malaria is the third cause of death among people infected with HIV, particularly pregnant women.

Effective integrated care of patients with malaria and HIV infection in regions where the two coexist is therefore an international health challenge.

### Relevance

Concurrent HIV infection and malaria during pregnancy is an emerging problem in mother and child health, with an estimated 1 million pregnancies per year among women with both infection and malaria. In 11 of the 43 countries of Africa in which malaria is endemic (Botswana, Burundi, Central African Republic, Ethiopia, Malawi, Mozambique, Rwanda, South Africa, Swaziland, Uganda and Zambia), the prevalence of HIV infection among pregnant women seen at antenatal visits varied from 10% to more than 25% [4,5]. The estimated proportion of malaria cases due to HIV-induced susceptibility during pregnancy is 4.8% (95% confidence interval, 3.8 to 5.8), representing 50,382 additional cases of malaria [6].

As the clinical and biological symptoms of malaria are enhanced by co-infection with HIV, pregnant women with HIV infection more often have episodes of malaria that evolve into complicated forms [7].

The World Health Organization (WHO) recommends that pregnant women in endemic regions undergo four antenatal examinations and receive two doses of sulfadoxine-pyrimethamine (SP) at each visit (directly observed treatment (DOT)), beginning at week 16 of pregnancy [8]. Steketee *et al*. [9] however, reported a persistently high rate of peripheral and placental parasitaemia after two doses of intermittent preventive treatment (IPT) with sulfadoxine-pyrimethamine (SP) in HIV-infected pregnant women. Filler *et al*. [10] reported that a monthly dose of SP was significantly more effective than two doses (prevalence of placental parasitaemia, 7.8% versus 21.5% in HIV-positive women and 2.3% versus 6.3% in HIV-negative women.

Ascertaining HIV serological status has become a prerequisite for better malaria prevention. The efficacy of co-trimoxazole in preventing *Plasmodium falciparum* malaria is well recognized. Thus, Mermin *et al.*[11] reported a reduction of 40 malaria episodes per 100 person-years in adults (other than pregnant women), and a clinical trial of co-trimoxazole versus SP among children in Bandiagara, Mali [12], showed a protective efficacy of 99.7%. Furthermore, several studies showed a good clinical and parasitological response to co-trimoxazole in treated children [13-16], and it has been reported to be safe in long-term use during pregnancy [17].

Therefore, preventive treatment with SP for all HIV-positive people (including pregnant women) who are receiving treatment containing co-trimoxazole is superfluous and is even contraindicated in light of its common side-effects (particularly severe skin reactions). Few studies, however, have described the efficacy of co-trimoxazole in preventing malaria among pregnant women, particularly in areas where the frequency of therapeutic failures with SP in cases of *P. falciparum* malaria is increasing.

The emergence of an increase in the frequency of resistance of *P. falciparum* to SP in many sub-Saharan African countries [18,19], including the Central African Republic [20,21], challenges the short-term usefulness of this drug combination in preventing malaria among pregnant women. The resistance is due to the accumulation of point mutations at various sites on the genes that code for dihydrofolatereductase (*dhfr*) and dihydropteroate synthase (*dhps*). The number of mutations correlates with the extent of *P. falciparum* resistance to SP *in vitro*. In studies carried out in Bangui, the prevalence of therapeutic failure was estimated to be 23.8% after 14 days of follow-up among children with uncomplicated malaria [20], while the resistance of *P. falciparum* to pyrimethamine *in vitro* was reported to be 38.3% [21]. The frequency of triple mutations in alleles Ile-51, Arg-59 and Asn-108 was 50.3% [22], and there was a strong positive correlation between the number of mutations and the resistance *in vitro* of *P. falciparum* strains [23]. The triple mutations that can occur in *dhfr* at Ile-51, Arg-59 and Asn-108 and the double mutation at Gly-437 and Glu-540, alone or in combination, are associated with therapeutic failure of SP [24].

Pregnancy and HIV infection increase the risk for emergence of mutated strains that are resistant to SP, partly because a wide variety of types and clones are found in parasitaemia among pregnant women (genetic diversity) [25], but also because the circulating biomass of *P. falciparum* has increased due to the HIV epidemic [26]. There is still concern about the possible development of cross-resistance of *P. falciparum* to both co-trimoxazole and SP because of their similar modes of action, although this hypothesis has not been proven [27,28]. A study of the response of wild parasites mutated in the *dhfr* and *dhps* genes to SP and co-trimoxazole *in vitro* showed partial cross-resistance to the two drugs [29]. Mutations in Arg-59 of *dhfr* and Gly-43 of *dhps* correlated with *P. falciparum* resistance to co-trimoxazole [15].

The current prevalence of malaria among women at first presentation to an antenatal clinic in Bangui is estimated to be 23% [30]. Since 2006, the national malaria programme has established administration of SP-IPT, with two doses for HIV-negative women and three doses for those who are HIV-positive [31].

In 2011, we assessed data from maternity centres of Bangui and found that more than 75% of pregnant women accepted HIV testing. This finding is in accordance with the recommended threshold of acceptance [32]. Our preliminary evaluation showed a relatively high rate of HIV infection (7.3%) among pregnant women. The profile of HIV serological status and rates of prevention of mother-to-child transmission (MTCT) are detailed in Table 1.

**Table 1:** Profile of HIV infection in pregnant women who accepted testing in Bangui, 2011

**Hospital**	**Number offered HIV testing**	**Number (%) accepted HIV testing**	**Number (%) positive**	**Number (%) who asked for result**	**Number receiving prevention of mother-to-child transmission**
Castors	612	461 (75.3)	34 (7.4)	28 (82.3)	23 (82.1)
Communautaire	1218	887 (72.8)	70 (7.9)	57 (81.4)	55 (96.5)
Amitié	716	602 (84.1)	38 (6.5)	36 (92.3)	30 (83.3)

To prevent opportunistic infections, co-trimoxazole is given to all women at WHO clinical stages 2, 3 and 4 and those at stage 1 with a CD4+ count <350/mm^3^ from the second trimester and throughout pregnancy [33].

In the Central African Republic, two treatment schemes are recommended for the management of HIV infections during pregnancy [32]:

•Antiretroviral treatment is offered to all pregnant women with severe or clinically advanced disease, regardless of their CD4+ count, and for those with a count of ≤350 cells/mm^3^, regardless of their symptoms. Treatment begins at any time during pregnancy. First-intention treatment should comprise zidovudine (AZT) plus lamivudine (3TC), with nevirapine (NVP) or efavirenz (EFV). Breastfed infants should receive NVP or AZT throughout breastfeeding.

•Women who are ineligible for antiretroviral treatment are given a preventive regime to reduce HIV MTCT: (i) AZT from week 16 of amenorrhoea, (ii) AZT, 3TC and NVP during labour and delivery and (iii) AZT and 3TC for 7 days after delivery. Breastfed infants receive a dose of NVP at birth and a daily dose from birth to 1 week before weaning. Infants who are not breastfed are given a single dose of NVP at birth, followed by one dose per day of NVP or AZT up to the age of 6 weeks.

### Hypothesis

Our main hypothesis is that co-trimoxazole is more efficacious than SP-IPT against placental parasitaemia, perhaps due to a higher plasma concentration of co-trimoxazole attained with daily doses. If we validate this hypothesis, co-trimoxazole could be recommended as prophylaxis for HIV-positive pregnant women, regardless of their CD4+ cell count. In this study, we will also test the hypothesis that the strains of *P. falciparum* isolated from HIV-positive pregnant women express more *dhfr* and *dhps* resistance markers.

## Methods/Design

### Aims

The main objective of this randomized trial is to confirm that co-trimoxazole is more efficacious than SP against placental parasitaemia (primary end-point) among HIV-positive pregnant women with a CD4+ count >350 cells/mm^3^.

We will also compare the two treatments with regard to adherence, the occurrence of adverse events, the frequency of maternal anaemia (hemoglobinaemia <10 g/dl), malaria episodes during pregnancy, abortions, stillbirths, prematurity (birth <37 weeks of amenorrhoea) and low birth weight (<2500 g), placental malaria, umbilical malaria transmission, and mother-to-child HIV transmission.

### Study design

The MACOMBA study (MAternity and COntrol of Malaria-HIV co-infection in BAngui) is a multicentre randomized controlled open-label trial to compare SP-IPT with co-trimoxazole. The study will be open-label, because it will be impossible to blind the pregnant women and health-care workers involved to which group the women are assigned. Furthermore, we seek to compare the two strategies under real-life conditions.

The trial will be performed by the Institut Pasteur of Bangui, supported by Institut Pasteur of Paris. The four main maternity hospitals in Bangui are involved in this study.

### Participants

HIV-seropositive pregnant women will be recruited in the four maternity clinics. All pregnant women attending maternity clinics will be offered an HIV test as part of the standard procedure of HIV counselling [34]. Inclusion in the study will be offered to all women presenting for their first visit between weeks 16 and 28 of amenorrhoea and with confirmed HIV infection. Women who arrive for their first antenatal visit before week 16 of pregnancy can be included at their second visit if they are eligible. Women who already know their HIV status can also be included if their CD4+ count is >350 cells/mm^3^ and they have no opportunistic diseases (WHO stage 2, 3 or 4). These women will not be re-tested for HIV, but their CD4+ count will be checked at this stage of inclusion in the study.

### Eligibility criteria

The inclusion criteria are age ≥18 years, HIV positivity; gestation of 16 to 28 weeks; CD4+ count >350 cells/mm^3^ and no sign of WHO stage 2, 3 or 4, agreement to attend all antenatal consultations for the study, willingness to adhere to all the requirements of the study (including voluntary counselling and testing for HIV) and signed informed consent.

The exclusion criteria are psychological instability that might interfere with compliance, hypersensitivity to sulfamides or dermatological disease (eczema, pemphigoid exanthema) that would increase the risk for severe reaction to the drugs being tested; severe anaemia (Hb <7 g/dl), known hepatic cardiac or renal disease, or any other severe disease.

### Recruitment and randomization

Potential study participants will be identified by midwives at antenatal clinics. Women eligible for participation in the study will be invited for additional counselling by a research midwife to ensure that they are fully informed orally and by written material about the nature of the study. After providing informed consent to participate in the study, the women will be randomly allocated to either the SP-IPT or co-trimoxazole group.

Randomization of women will be centralized and stratified according to maternity clinic and gravidity (primigravidae versus multigravidae). For each maternity clinic, two random lists for SP-IPT and co-trimoxazole in a ratio 1:1 will be generated for primigravidae and multigravidae, with the R software (version 2.14.1). Once a pregnant woman is confirmed to be eligible for the study, the field investigator will telephone the coordination staff at the Institut Pasteur of Bangui to indicate the gravid rank, and the site staff will assign women to a treatment arm according to the randomization list, respecting the chronological order of inclusion.

### Intervention

#### Study drugs

The drugs to be used in this study will be supplied by the International Dispensary Association in the Netherlands. Doses of SP-IPT and co-trimoxazole will be administered from week 16 to the end of pregnancy. Three doses of SP will be administered under direct observation, equivalent to 25 mg of sulfadoxine and 1.25 mg of pyrimethamine per kilogram of body weight. The first dose will be given between weeks 16 and 28 of amenorrhoea, the second 1 month later and the third 1 month after the second.

A daily dose of one co-trimoxazole tablet (containing 160 mg trimethoprim and 800 mg sulfamethoxazole) will be given until delivery [17]. Compliance will be evaluated at each antenatal visit, by questioning the women about the doses they have taken and by asking them to bring to the visit all remaining tablets.

All women will be given iron supplementation (200 mg) and folic acid (0.4 mg) and will receive an insecticide-impregnated bed net (donated by the Total Group in Central African Republic and the National Malaria Programme).

During follow-up, any suspected malaria episode will be followed up immediately with parasitological evaluation (blood smear). Treatment with quinine at a dose of 24 mg/kg body weight will be given at three doses per day 8 hourly apart for 7 days. The tablet form of quinine will be given in the absence of severe clinical signs. If the malaria episode is associated with severe clinical symptoms, quinine (perfusion in 10% serum glucose) will be administered parenterally at a dose of 24 mg/kg body weight per day until clinical evaluation shows that oral quinine can be taken. Patients who experience adverse effects of quinine will be treated with artemether or artemisinin combined treatment according to national guidelines.

**Figure 1:**
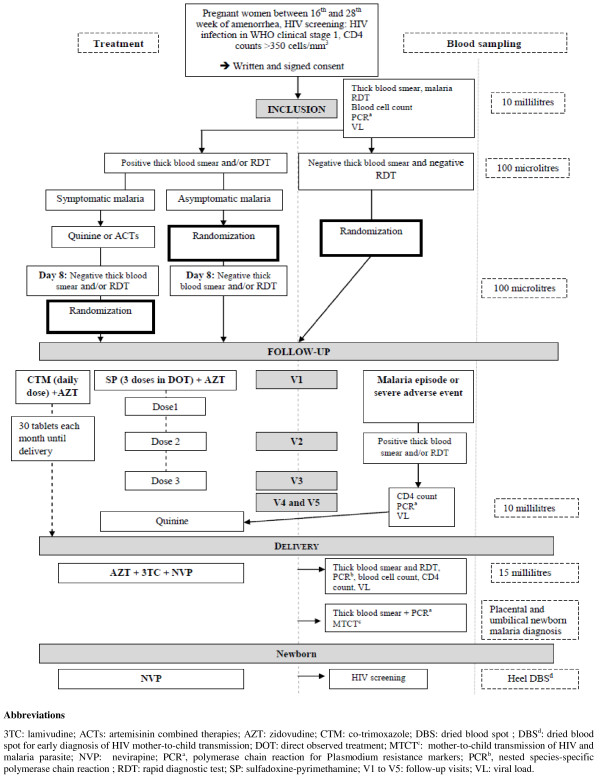
**MACOMBA trial flowchart.** 3TC: lamivudine; ACTs: artemisinin combined therapies; AZT: zidovudine; CTM: co-trimoxazole; DBS: dried blood spot; DBS^d^: dried blood spot for early diagnosis of HIV mother-to-child transmission; DOT: direct observed treatment; MTCT^c^: mother-to-child transmission of HIV and malaria parasite; NVP: nevirapine; PCR^a^, polymerase chain reaction for *Plasmodium* resistance markers; PCR^b^, nested species-specific polymerase chain reaction; RDT: rapid diagnostic test; SP: sulfadoxine-pyrimethamine; V1 to V5: follow-up visits; VL: viral load.

A woman who fulfils all the inclusion criteria but is found to have symptomatic malaria parasites at recruitment will be treated for the malaria episode and will not be included in the study. She will be reconsidered for inclusion later, when she is no longer parasitaemic. All women with asymptomatic malaria parasites at baseline will be included and randomized to either the SP-IPT or co-trimoxazole group. If symptomatic malaria or persistence of asymptomatic malaria parasitaemia is seen 8 days later, the woman will be given curative treatment, as stated above (Figure 1).

### Management of adverse events during follow-up

All women included in this study will undergo regular clinical surveillance (once a month) and will be encouraged to notify the doctor involved in the study of any adverse events. The adverse effects attributed to SP-IPT and co-trimoxazole will be addressed according to cutaneous toxicity, classified according to the grades of the French National Agency for AIDS Research. Symptomatic medications will be given, and the study may be interrupted at the discretion of the clinicians.

All adverse events associated with HIV infection or antiretroviral treatment will be managed by the national service for prevention of MTCT.

All women whose HIV infection evolves to clinical stage 2 or who have a CD4 count interval of ≤350 per mm^3^ will be sent to the centre for antiretroviral treatment at the Hôpital Communautaire or Hôpital de l’Amitié with a referral to the service for infectious diseases.

### Data collected

At baseline, the following data will be collected: socio-demographic characteristics, clinical data, obstetric history, gestational age estimated from the date of the last menstrual period and/or measured by uterine fundal height, and biological end-points (CD4 count, HIV viral load (VL), malaria diagnosis, and blood cell count). During follow-up, standard clinical examinations will be performed monthly. Adverse events will be recorded and any suspected malaria episode will be investigated by laboratory testing. Blood will be taken from women presenting with a malaria episode, and CD4 count and HIV VL will be determined.

Polymerase chain reaction (PCR) for malaria diagnosis and identification of resistant strain markers (*dhfr* and *dhps*) will be performed for all episodes of *Plasmodium* infection.

At delivery, the women’s blood will be analysed for CD4 count, HIV VL, blood cell count, and malaria diagnosis (thick smear analysis and rapid diagnostic test for malaria and nested species-specific PCR). At this point, MTCT of HIV and possible passage of the malaria parasites though umbilical blood circulation will be assessed.

### Blood drawing

Venous blood will be collected in ethylenediaminetetraacetic acid (EDTA) minicollect tubes at inclusion, at scheduled and unscheduled antenatal clinic visits if a malaria infection is diagnosed, and after delivery. These blood samples will be tested for blood cell counts and HIV VL estimation. Finger pricks for thick and thin blood smear preparation and antigen Pf/Pan-SD Bioline™ rapid diagnostic testing will be performed for any suspected episodes of malaria during follow-up. Maternal blood will be also collected on filter paper for *Plasmodium* DNA extraction. Early screening of HIV and malaria parasites transmission in infants will be determined from heel-prick and umbilical blood respectively. Placental blood will be collected immediately after delivery. For this procedure, a block of tissue (1 × 1 × 1 cm) will be excised from the basal side of the placenta, resulting in a large pool of intervillous blood at the excision site. The placental tissues will be pressed to prepare thin and thick blood smears (Figure 1).

### Laboratory analysis

Malaria rapid diagnostic tests and thick smear analyses will be performed at the study sites, while other laboratory analyses will be performed at the Institut Pasteur of Bangui.

CD4+ cells will be counted by cytometry (FACSCalibur™, Becton Dickinson, San Jose, CA, USA), and blood cell counts will be conducted by the ABX PENTRA 60 (HORIBA ABX Diagnostics, Irvine, CA, USA).

HIV VL will be estimated at the Institut Pasteur of Bangui with the ABI PRISM 7000 (Generic HIV kit; Biocentric, Bandol, France).

Generic HIV DNA Cell kits (Biocentric, Bandol, France) will be used for PCR to assess maternal-child transmission of HIV infection.

The antigen Pf/Pan-SD Bioline™ rapid diagnostic test will be conducted according to the manufacturer’s guidelines. Thick blood smears will be air-dried, stained with 4% Giemsa and analysed under a light microscope (× 100 oil immersions) to detect asexual forms of *P. falciparum*. Nested species-specific PCR will be performed to screen maternal and placental blood samples for malaria parasites as described previously [35]. The greater sensitivity of PCR than of rapid diagnostic tests and microscopy has been proven [36,37].

Molecular studies to identify *Plasmodium* resistance markers to pyrimethamine and sulfadoxine (*dhfr* and *dhps*) will be performed on blood samples with a positive malaria result. The genotypes *pfdhfr 51*, *59* and *108* and *pfdhps 437* and *540* will be determined by nested PCR [38].

### Outcome measures

The primary end-point of the trial is placental parasitaemia at delivery. Data on this variable will be coded as positive or negative results. Other outcome measures are adherence to co-trimoxazole treatment, adverse events related to co-trimoxazole and SP-IPT, the incidence of malaria episodes during pregnancy and complications due to malaria: anaemia (hemoglobinaemia <10g/dl), abortion (delivery <28 weeks of amenorrhoea), stillbirth, prematurity (birth <37 weeks of amenorrhoea) and low birth weight (<2500 g body mass), mother-to-child HIV transmission and umbilical blood parasitaemia. At delivery, maternal, placental and newborn umbilical blood will be sampled to detect the asexual form of *Plasmodium*.

### Statistical considerations

#### Sample size

On the basis of previous studies [10,17,39,40], we anticipate that there will be 6.2% placental malaria infections in the co-trimoxazole group and 16.8% in the SP-IPT group. We will therefore randomize 300 women (type I error of 0.05, power of 80%, one-sided test), of whom 150 will be in the experimental group and 150 in the control group.

### Statistical analysis

The primary end-point will be analysed as intention-to-treat; deaths and women lost to follow-up will be considered failures. Analysis of the ‘per protocol’ population will be conducted among women who complete the study until delivery and undergo the recommended initial treatment.

The data will be recorded in an ACCESS™ database (Microsoft Corp., Redmond, WA, USA) and will be analysed with STATA™ software (version 12; Statacorp, College Station, TX, USA). The analyses will be carried out after stratification according to the maternity clinic and gravidity (primigravidae versus multigravidae). The chi-squared test or Fisher’s exact test will be used to compare categorical variables between the two groups, including the number of positive placental malaria infections (primary end-point), cumulated incidence of malaria episodes during pregnancy, proportion of parasite strains with *dhfr* mutations, maternal anaemia and maternal morbidity or mortality. Student’s *t* test or the Mann-Whitney test will be used to compare continuous variables in the two arms. A logistic regression model will be used, with adjustment for maternity clinic, gravidity, age, CD4 count and anaemia to compare the two groups of women for the number of placental malaria infections, malaria episodes during pregnancy, and identified resistance.

### Ethical and legal considerations

The National Ethics Committee of the Central African Republic and the Clinical Research Committee of the Institut Pasteur in Paris approved the study protocol.

## Discussion

In this trial, we seek to confirm that co-trimoxazole is operationally suitable to replace SP-IPT in order to prevent malaria among pregnant women infected with HIV in the Central African Republic. Studies of alternatives to SP in preventing malaria during pregnancy have been designed in Benin [41,42]. The MACOMBA project will provide further information about the suitability of co-trimoxazole for malaria prevention among HIV-infected pregnant women, regardless of their CD4 count. It may result in better management and therefore better outcomes for infected pregnant women. The women included in this study will receive adapted therapeutic care and regular, standardized biological follow-up, which will improve their quality of life and ensure satisfactory pregnancies. At the community level, the project will sensitize the population, offering them a better understanding of the risks associated with malaria, both for pregnant women and for the infants. It should also strengthen prevention of MTCT of HIV, which remains relatively high (11%) in the Central African Republic (Ministry of Public Health, Population and AIDS Control, *unpublished data*). If the frequency of mutations in malaria parasites is found to be higher in the SP arm of this study, all seropositive women will be offered treatment with co-trimoxazole. The results of this study will be communicated to health policy-makers and experts who make recommendations about malaria prevention among HIV-positive women.

## Trial status

This study is currently being implemented.

## Competing interests

The authors declare that they have no competing interests.

## Authors’ contributions

AM, MV and MK were involved in the conception and design of the study. AS, ES, SG, GGBB, SM, AB, JMM, OS and LL have made substantial contributions to the design and are involved in the conduct of the study. AM, MV and MK drafted the manuscript. All authors mentioned in the manuscript are members of MACOMBA study group. They read and approved the final manuscript.
